# Network analysis of the proteome and peptidome sheds light on human milk as a biological system

**DOI:** 10.1038/s41598-024-58127-2

**Published:** 2024-03-30

**Authors:** Pieter M. Dekker, Sjef Boeren, Edoardo Saccenti, Kasper A. Hettinga

**Affiliations:** 1 Food Quality and Design Group, Wageningen University and Research, Wageningen, 6708 WE The Netherlands; 2 Laboratory of Biochemistry, Wageningen University and Research, Wageningen, 6708 WE The Netherlands; 3 Laboratory of Systems and Synthetic Biology, Wageningen University and Research, Wageningen, 6708 WE The Netherlands

**Keywords:** Proteome, Peptides, Data integration, Enzyme mechanisms, Proteases, Biochemistry, Systems biology, Proteome informatics, Proteolysis, Proteomics

## Abstract

Proteins and peptides found in human milk have bioactive potential to benefit the newborn and support healthy development. Research has been carried out on the health benefits of proteins and peptides, but many questions still need to be answered about the nature of these components, how they are formed, and how they end up in the milk. This study explored and elucidated the complexity of the human milk proteome and peptidome. Proteins and peptides were analyzed with non-targeted nanoLC-Orbitrap-MS/MS in a selection of 297 milk samples from the CHILD Cohort Study. Protein and peptide abundances were determined, and a network was inferred using Gaussian graphical modeling (GGM), allowing an investigation of direct associations. This study showed that signatures of (1) specific mechanisms of transport of different groups of proteins, (2) proteolytic degradation by proteases and aminopeptidases, and (3) coagulation and complement activation are present in human milk. These results show the value of an integrated approach in evaluating large-scale omics data sets and provide valuable information for studies that aim to associate protein or peptide profiles from biofluids such as milk with specific physiological characteristics.

## Introduction

Proteins in human milk have a wide variety of biological functions, ranging from nutrition to immune modulation^1^. Their synthesis can occur in the mammary epithelial cells (MECs), which is the case for the major milk proteins, such as the caseins and $\alpha$-lactalbumin (LALBA, also known as ALA)^2^. Other proteins are believed to be synthesized in other parts of the body and are subsequently transferred towards and through the MECs^3^. Shared location of synthesis, shared mechanism of transfer, or functioning in the same biological pathways can result in interdependencies between proteins^4^.

Parts of the amino acid sequence of proteins can, once detached from the original sequence, exert a completely different biological and biochemical activity. This detachment can occur during proteolytic degradation, resulting in peptides and free amino acids. In human milk, proteolytic degradation starts already when milk is secreted into the alveolar lumen^5^ and is due to proteolytic systems comprising proteases, protease activators, and protease inhibitors^6^. Active proteases, such as plasmin (PLG) and kallikrein, hydrolyze peptide bonds between amino acids in the protein sequence, disrupting the protein’s primary structure^7^.

It is known that peptides play a considerable role in many cellular processes in the body, for example, acting as hormones, cytokines, or growth factors^8,9^. Nevertheless, the role of peptides in human milk is not entirely understood yet. Some peptides can exert specific bioactivities, such as immunomodulatory, antimicrobial, antioxidative, or angiotensin-converting enzyme (ACE) inhibitory effects^10,11^. These bioactivities could be beneficial for protecting the mammary gland against infection but also have health benefits for the breastfed infant^12^. Although proteolytic degradation in the digestive system could result in the breakdown of bioactive peptides, specific peptide sequences might be protected against, or resistant to, further proteolytic degradation. In addition, new bioactive peptides may be formed upon enzymatic digestion in the infants’ gastrointestinal tract from either intact proteins or larger peptides^11^.

To date, several studies have investigated the human milk peptidome from a mechanistic perspective^13,14^, focusing on cleavage patterns and protease specificity. Although this has provided valuable insights into the human milk peptidome, much still needs to be discovered. Since peptides are a product of larger peptides or proteins, and since the proteolytic systems themselves are part of the proteome, it is expected that relationships exist between the proteome and peptidome. Analysis of these relationships in an integrated approach is an important step in increasing knowledge about the proteolytic activity in human milk.

This study aimed to investigate associations among proteins, among peptides, and between proteins and peptides in human milk. Proteomics and peptidomics profiles from 297 human milk samples, obtained using LC-MS/MS, were subjected to network analysis using Gaussian graphic modeling (GGM), and observed associations were discussed. The rationale behind this approach is that the associations observed in the GGM network can provide information about the biological function of the proteins and peptides and how they are formed or end up in the milk^15,16^. The resulting pairwise partial correlations enable a distinction between indirect and direct associations by adjusting for the contribution of all remaining variables^17^. The importance of studying human milk as a unique biological system has recently been emphasized by Christian et al. in a comprehensive perspective^18^. Human milk is the sole source of nutrition for infants, in a vulnerable period that is critical for development and health. Yet, little is currently known about the functionality of nutritional, biological, and immunological pathways of its components driving growth, development, and health in early life^18^. This research thereby provides an alternative way to integrate and interpret large-scale multi-omics data sets and shows the added value of studying associations in the human milk protein and peptide profile.

## Results and discussion

### Mass spectrometry analysis of proteins and peptides in human milk

The LC-MS/MS analysis resulted in the identification of 1690 proteins and 9192 peptides originating from 48 precursor proteins.

After filtering the data on the requirement of identification in more than half of the samples, 480 proteins (Supplementary Table S1) and 1455 peptides (Supplementary Table S2) remained, with the peptides still originating from 48 precursor proteins (Supplementary Table S3). The relative contribution of the precursor proteins to the peptidome showed that the majority of the peptides originated from $\beta$-casein (38.5%), polymeric immunoglobulin receptor (PIGR) (10.5%), and butyrophilin subfamily 1 member A1 (BTN1A1, also known as BTN) (8.5%), a similar pattern as found in previous studies^19,20^.

### Network analysis

A protein and peptide association network was inferred by the generation of GGMs. Edges were drawn in the network if partial correlations were significant (local fdr < 0.1). This resulted in an initial network containing 16,961 edges connecting 1895 nodes. This network summarizes the web of associations and interactions (between 448 protein and 1447 peptides) inferred from the abundance profiles of proteins and peptides. Molecular functions of proteins are often carried out through interaction with other proteins^21^. Similarly, peptides are essential players in human physiology, originating from, and interacting with, a diverse range of proteins and actively participating in numerous cellular functions^22,23^. For these reasons, the observed associations between proteins and peptides can be viewed as proxies for the study of underlying molecular mechanisms. Only 16,961 edges (corresponding to 0.9% of all possible edges between proteins and peptides) were found to be statistically significant, indicating a rather sparse and disconnected network as often observed in protein-protein interaction studies^24^.

**Figure 1:**
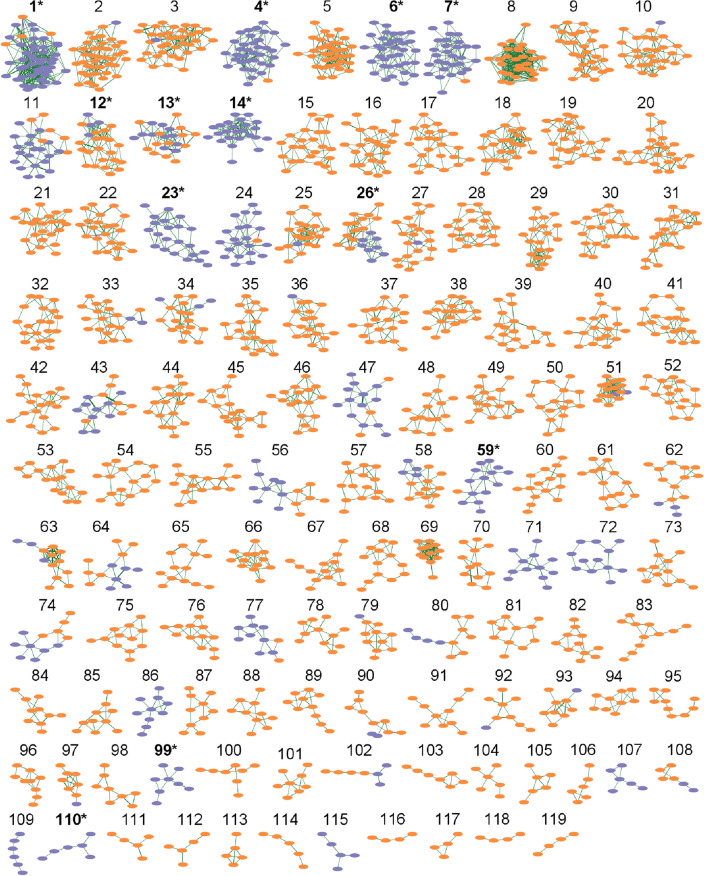
Network representation of associations between proteins and peptides. The network is constructed by calculation of Gaussian graphical models (GGMs) and subsequent clustering with the Leiden community detection algorithm. Purple nodes represent proteins, and orange nodes represent peptides. The thickness of the edges is proportional to the partial correlation coefficients from the GGMs. Clusters with a significant overrepresentation of Gene Ontology (GO) annotation have a label in bold and are indicated with *. A high-resolution version of this figure, which includes labeling of the individual nodes, is provided as Supplementary Fig. S1.

Using the Leiden community detection algorithm (Fig. 1, Supplementary Fig. S1), 119 clusters were found containing proteins/peptides sharing similar patterns of association: 35 included both proteins and peptides, whereas 11 clusters only comprised proteins, and 73 clusters only comprised peptides. The biological relevance of these clusters is discussed in the next sections. Most connections (95.4%) were observed among features from the same data set, that is, cross-associations among proteins and among peptides.

### Associations among proteins

GO annotation of proteins was used to investigate the overrepresentation of annotations in clusters of associated proteins. An overview of the 12 clusters for which the proteins showed significant overrepresentation of annotations can be found in Table 1. We present a discussion of a selection of these clusters.

**Table 1:** Clusters with Gene Ontology (GO) overrepresentations.

Cluster	Gene Ontology domain^a^	Gene Ontology annotation	Adjusted *p* value	Associated proteins
1	CC	Pentameric IgM immunoglobulin complex	$1.60\times 10^{-2}$	IGHM, IGJ
4	CC	Blood microparticle	$1.18\times 10^{-7}$	A1BG, CFH, C9, HPX, SERPINC1, AHSG, PLG, SERPINF2, ORM1, ORM2, HP, ALB, AFM, F2, CP, IGHG4, ITIH4
	BP	Acute inflammatory response	$2.62\times 10^{-6}$	ORM2, HP, SERPINC1, SERPINA1, AHSG, F2, ITIH4, ORM1, SERPINF2
6	CC	Bounding membrane of organelle	$6.40\times 10^{-6}$	STOM, RAB1A, RHOA, SAR1A, SNAP23, PLIN3, RAB6A, CD36, RAP1B, CNP3, MGAT132, STX3, RAB18, EHD4, TLR2, GNB1, YKT6, ARF1, GLIPR2
	MF	Hydrolase activity, acting on acid anhydrides, in phosphorus-containing anhydrides	$1.81\times 10^{-3}$	RAP1B, ABCG2, RHOA, RAB18, RAB1A, ENTPD3, RALB, SAR1A, GNB1, ARF1, RAB6A
7	CC	Extracellular space	$7.52\times 10^{-3}$	CD59, TIMP1, B4GALT14, LALBA, VTN, SERPING1, FAM3C, KLK6, TCN1, MIF, SPINT1, CSN2, GPX3, CSN3, DAG1, LGALS3BP, LTF, CA6, LRG1, VWA1, CD14, RNASET2
12	CC	Fibrinogen complex	$1.25\times 10^{-3}$	FGA, FGB, FGG
	BP	Regulation of vasoconstriction	$5.40\times 10^{-4}$	FGA, FGB, FGG
13	CC	High-density lipoprotein particle	$2.42\times 10^{-9}$	CLU, APOE, APOA1, APOA2, APOA4, APOB, PLTP
	MF	Intermembrane cholesterol transfer activity	$1.07\times 10^{-8}$	APOE, APOA1, APOA2, APOA4, APOB, PLTP
	BP	Sterol transport	$2.08\times 10^{-8}$	CLU, APOE, APOA1, APOA2, APOA4, APOB, PLTP
14	CC	Lysosome	$1.57\times 10^{-6}$	CTSB, NAGLU, LGMN, CTSD, FUCA1, ARSA, CTSZ, PSAP, HEXB, GRN
	BP	Granulocyte activation	$1.31\times 10^{-2}$	CTSB, PSMA5, 5, ,CTSD, CHI3L1, HSPA6, GNS, PDXK, CTSZ, PSAP, DPP7, FUCA1, ARSA, GRN, HEXB
23	CC	Endoplasmic reticulum lumen	$3.36\times 10^{-4}$	B2M, HSPA5, HSP90B1, P4HB, PDIA3, RCN1, HYOU1, CALR, TGOLN2, CALU, SUMF2, SDC2
	BP	Response to endoplasmic reticulum stress	$1.24\times 10^{-3}$	HSPA5, HSP90B1, P4HB, PDIA3, HYOU1, CALR, MAN1A1
26	CC	Microvillus	$1.14\times 10^{-4}$	RDX, MSN, EZR, SLC9A3R1
	BP	Regulation of early endosome to late endosome transport	$1.35\times 10^{-3}$	RDX, MSN, EZR
59	BP	ESCRT complex disassembly	$3.96\times 10^{-2}$	CHMP1B, CHMP2A, IST1
99	MF	Transcription coactivator activity	$2.58\times 10^{-2}$	ACTN4, PARK7, ACTN1
110	CC	Ribosomal subunit	$1.32\times 10^{-3}$	RPS16, RPS18, RPL3, RPS3A, RPS4X
	MF	Structural constituent of ribosome	$1.89\times 10^{-3}$	RPS16, RPS18, RPL3, RPS3A, RPS4X
	BP	Establishment of protein localization to endoplasmic reticulum	$1.40\times 10^{-3}$	RPS16, RPS18, RPL3, RPS3A, RPS4X

Shown are all protein clusters with significant (adjusted *p* < 0.05) overrepresentation of GO annotations as indicated in Fig. 1. Gene names are used to abbreviate the protein names.

^a^*BP* biological process, *MF* molecular function, *CC* cellular component.

A significant overrepresentation was found for Cluster 1 of proteins annotated with pentameric immunoglobulin M (IgM) complex (adjusted *p* = 0.016). Nevertheless, it is important to note that annotation for molecular function was only available for 6 out of the 44 proteins in this cluster. This is because most of these proteins are variable regions of immunoglobulins (Igs), for which GO annotation is unavailable. Besides the variable regions, this cluster also comprises the heavy constant regions of IgM and IgA, as well as the Ig J chain, which links multimeric IgA and IgM. Therefore, the associations between heavy chains, light chains, J chain, and variable regions indicate their physical relation as substructures of antibodies. In addition, the associations between IgA and IgM also indicate their common origin, since IgA and IgM in milk are produced mainly in the plasma cells in the mammary tissue^25^. Subsequent transepithelial transport of these proteins mediated by PIGR results in their secretion in milk^26^. Among the determinants of the human milk immunoglobulins are, e.g., maternal vaccination, smoking, psychological stress, and maternal or infant infection^27^.

Cluster 4 shows a significant overrepresentation (*p* = $1.18\times 10^{-7}$) of proteins commonly located in blood microparticles, which are microvesicles found in the blood. The cluster comprises 35 proteins, of which 17 are annotated as a component of blood microparticles. Among these are, for example, serum albumin (ALB), the major milk protease PLG, and protease inhibitors (4 serine protease inhibitors (SERPINs) and an inter-$\alpha$-trypsin inhibitors (ITIs)). Immunoglobulin G (IgG) is present in Cluster 4 as well. It is known that IgG in milk mainly originates from blood serum^25^, and the association with the blood proteins in Cluster 4 supports this.

It is generally assumed that PLG, which has an important role in blood coagulation, is blood-derived and transported into the milk from the systemic circulation^28^. In addition, a recent study has shown that one of the SERPINs in this cluster, SERPINA1 (also referred to as $\alpha$_1_-antitrypsin (A1AT)), is synthesized in the liver and enters the milk via direct transmission from the systemic circulation^3^. Considering the overrepresentation of proteins typically found in blood with functional characteristics in blood coagulation, it can be hypothesized that this cluster represents proteins originating from the systemic circulation, all being passively transported via a transcellular or paracellular pathway through the mammary epithelium. Furthermore, changes in the abundance of these proteins indicate a change in the relative importance of this transport. In reviewing the blood-milk-barrier (BMB) in cows, Wellnitz et al. refers to three possible mechanisms causing an increase in transport through the BMB^29^. First, the barrier can be impaired during inflammation when changes take place in the epithelial barrier. Second, the BMB can be impaired during lactation by oxytocin release or the physical force of suckling. Third, prolonged milk stasis is also known to impair tight junction permeability. An increase of the proteins in Cluster 4 might therefore indicate an impaired BMB due to one of the mechanisms described by Wellnitz et al.^29^.

Proteins that are known to be part of the milk fat globule membrane (MFGM) were found in Cluster 6^30^. Within the epithelial cell, milk fat globules (MFGs) are surrounded by a single-layer membrane that comprises proteins, such as lactadherin (MFGE8), cluster of differentiation 36 (CD36), mucin 1 (MUC1), and BTN1A1. These proteins are believed to support the MFG in moving towards and binding to the apical plasma membrane^31^, which forms the outer bilayer of the MFGM after secretion. The clustering of the typical MFGM proteins (Cluster 6) and the GO overrepresentation of the bounding membrane of organelle as a cellular component (*p* = $6.40\times 10^{-6}$) confirms that these proteins are related to the membrane and thus have a common origin. It is known that one of the determinants of the fat content of human milk is the produced milk volume^32^. Fat content itself is related with MFG size, where a higher fat content results in larger MFGs^33^. These larger MFGs are easier to disrupt, resulting in an increased amount of dissociated MFGM and consequently MFGM proteins in the milk.

Cluster 7 shows an overrepresentation of proteins located in the extracellular space (*p* = $7.52\times 10^{-3}$). Among the proteins in this cluster are the major milk proteins, including both caseins and whey proteins, such as LALBA, $\beta$-casein, $\kappa$-casein, and lactoferrin (LTF, also known as LF). It is known that these proteins are synthesized in the mammary gland^2^, a process that is regulated by lactogenic hormones (insulin, prolactin, and glucocorticoids), and amino acids^34^. Therefore, considering the strong associations between these proteins, it can be assumed that their expression is related to activation of the translation machinery and can be distinguished from the other proteins. Interestingly, this Cluster 7 does not include $\alpha$_S1_-casein. Unlike the other casein subunits, $\alpha$_S1_-casein does not decrease over lactation like $\beta$-casein and $\kappa$-casein^35^. In addition, it was found that this protein is not uniquely expressed in the mammary gland but also in monocytes^36^. The abundance of this protein in milk might therefore be more dependent on other factors than hormonal regulation.

Cluster 13 shows an overrepresentation of lipoprotein particles as the cellular location of the proteins (*p* = $2.42\times 10^{-9}$). The cluster comprises 6 different apolipoproteins, including apolipoproteins A1, A2, A4, B100, D, and E. It was shown in a study with mice that lipoprotein particles could be transferred from serum, deliver cholesterol in the MEC, and be secreted into the milk^37^. Considering the overrepresentation of apolipoproteins, the associations found in Cluster 13 might be an indicator of this mechanism. To date, no research has been done to investigate the cause of variation of these proteins in milk. However, it might reflect the extent of cholesterol transport in the mammary gland, which is known to be related with lactation stage and maternal diet^38,39^.

Cluster 14 comprises, amongst others, 3 cathepsins (B, D, and Z), progranulin (GRN), *N*-acetylglucosamine-6-sulfatase (GNS), and legumain (LGMN). These are proteins typically found in the lysosomal lumen^40^, which is also revealed by the GO overrepresentation of the lysosome as a cellular component for this cluster (*p* = $1.57\times 10^{-6}$). In addition, the strong associations observed between these proteins suggest that these proteins are released into the alveolar lumen through a common mechanism such as lysosomal exocytosis^41^. Lysosomes play an important role in cellular homeostasis, development, and aging. This role therefore points to lactation stage being the driving factor for the abundance of these proteins in the milk, which is in line with Watson et al., who points to the importance of lysosomal enzymes in mammary gland involution^42^.

The proteins ezrin (EZR), radixin (RDX), and moesin (MSN) form together the ERM protein family. These ERM proteins can bind with the Na(+)/H(+) exchange regulatory cofactor (SLC9A3R1, also known as NHERF1), and it is known that both ERM and SLC9A3R1 can act as a crosslinker between the actin cytoskeleton and cell membranes by interaction with the intracellular domain of the apical membrane protein podocalyxin (PODXL)^43,44^. Together, these proteins play an essential role in tissue integrity^45^. The association of these proteins in Cluster 26 suggests a loss of apical membrane from the MECs. Surprisingly, these proteins do not cluster with the typical MFGM proteins (Cluster 6), even though the outer bilayer of the MFGM is formed from the apical membrane. This suggests that the apical membrane found in human milk does not originate only from the MFGM. Explanations for this can be ongoing cell renewal of the MECs, apoptosis, or even the frozen storage of the samples, which results in damaging cells present in the milk and, consequently, a release of parts of the apical membrane. A study carried out by Qu et al. shows that frozen storage results in increased levels of, amongst others, EZR, MSN, and SLC9A3R1 in milk^46^.

Cluster 110 shows an overrepresentation of ribosomal constituents. Very little is known about why ribosomal proteins are present in milk. They might originate from exosomes, apoptosis of MECs, or intact or damaged cells present in the milk. Nevertheless, their association shows that their levels in milk depend on similar driving factors and possibly share the same secretion mechanism or origin.

Overall, results indicate that the abundance of the majority of the proteins in human milk depends primarily on the pathway of entering the milk.

### Associations among peptides

Our results show that clusters with peptides often comprise peptide ladders, differing only a few amino acids from the neighboring peptides (Table 2, Supplementary Table S4). The peptide ladders, which were found in peptide clusters, are presumably formed by aminopeptidases, which cleave a single amino acid off a peptide sequence (exoproteolysis). Several proteins with aminopeptidase activity were identified in the proteomics data of this study, which are in order of average abundance: dipeptidyl peptidase 2 (DPP7), cytosol aminopeptidase (LAP3), aminopeptidase B (RNPEP), aminopeptidase N (ANPEP), and leukotriene A-4 hydrolase (LTA4H). Although not all these aminopeptidases have been identified before in human milk, the activity of aminopeptidases in human milk has been evidenced^47^. The strong association observed between peptides of a peptide ladder suggests that this type of proteolytic degradation occurs in an abundance-dependent manner where the formation of a peptide depends on the abundance of its precursor.

**Table 2:** Overview of peptide ladders, i.e., peptides with partly overlapping sequences.

Cluster	UniProt ID	Protein name	Sequence range covered	Number of peptides within sequence range	Average peptide length
2	P01833	Polymeric immunoglobulin receptor	610*–643	9	18.0
3	Q13410	Butyrophilin subfamily 1 member A1	504–526	19	14.9
5	P07498	$\kappa$-Casein	79*–109*	22	17.4
8	Q13410	Butyrophilin subfamily 1 member A1	489–526	29	17.3
9	P05814	$\beta$-Casein	89–119*	7	17.6
10	P05814	$\beta$-Casein	86–161	20	19.2
12	P02671	Fibrinogen alpha chain	600*–629	11	21.2
15	P01833	Polymeric immunoglobulin receptor	598*–643	21	20.5
17	P05814	$\beta$-Casein	168–221	8	14.1
18	P05814	$\beta$-Casein	34*–54*	17	15.8
19	P01833	Polymeric immunoglobulin receptor	604*–640	13	17.5
20	P01833	Polymeric immunoglobulin receptor	607–633	6	15.7
25	P12272	Parathyroid hormone-related protein	37*–56*	8	17.1
25	P12272	Parathyroid hormone-related protein	90*–126*	6	15.0
29	P01833	Polymeric immunoglobulin receptor	572*–618	17	18.7
32	Q13410	Butyrophilin subfamily 1 member A1	68*–94*	12	16.6
35	P01833	Polymeric immunoglobulin receptor	605–643	8	16.2
36	P01833	Polymeric immunoglobulin receptor	624–648	6	21.7
42	P05814	$\beta$-Casein	16–40*	18	16.9
44	P01833	Polymeric immunoglobulin receptor	622–647	10	21.3
45	P05814	$\beta$-Casein	114*–132*	5	14.4
45	P05814	$\beta$-Casein	151–175*	7	14.4
46	P05814	$\beta$-Casein	16–40*	16	17.7
49	P05814	$\beta$-Casein	196–226	12	13.5
50	P05814	$\beta$-Casein	84–111*	8	22.0
51	P0C0L5	Complement C4-B	1429*–1449	9	17.0
52	P47710	$\alpha$_S1_-casein	26–51*	15	20.5
53	P05814	$\beta$-Casein	88–113*	16	20.7
54	P05814	$\beta$-Casein	99–223	10	19.7
55	P05814	$\beta$-Casein	145–167	12	13.8
57	P15941	Mucin-1	1207–1243	14	16.1
63	Q14512	Fibroblast growth factor-binding protein 1	27*–51*	6	21.7
65	P47710	$\alpha$_S1_-Casein	16–49	6	16.2
66	P10451	Osteopontin	204*–246	9	20.7
68	P10451	Osteopontin	176*–192	7	13.0
69	P05814	$\beta$-Casein	34*–61	12	18.2
73	P05814	$\beta$-Casein	170–194	7	16.1
75	P05814	$\beta$-Casein	104*–124	9	15.4
76	P19835	Bile salt-activated lipase	21–39*	8	16.4
81	P05814	$\beta$-Casein	21–40*	8	13.8
82	P47710	$\alpha$_S1_-casein	52*–68*	6	15.2
83	P05814	$\beta$-Casein	104*–121	11	13.0
87	P05814	$\beta$-Casein	97–120	9	16.3
89	P10451	Osteopontin	169*–203*	8	16.9
95	P05814	$\beta$-Casein	85–110	7	19.1
97	P05814	$\beta$-Casein	202–226	6	21.8
98	P05814	$\beta$-Casein	34*–58	7	21.4

Listed are all clusters comprising 5 or more overlapping peptides. Protein and peptide details listed are regarding the peptide ladder observed within the cluster. Rows are sorted on cluster id.

^∗^Cleavage position with the specificity matching the protease plasmin.

Before cleavage of larger peptides by aminopeptidases is possible, initial proteolytic degradation of proteins must occur (endoproteolysis). It has been suggested that endogenous proteases, especially PLG, carry out such proteolysis^13^. PLG is a highly specific protease that hydrolyzes the peptide bond between lysine (K) or arginine (R) in the P1 position and any other amino acid in the P1’ position. This specificity matches with several outer C-terminal or N-terminal positions of the peptide ladders observed in the clusters (Supplementary Table S4). Further allocation of proteases to endoproteolytic cleavage sites remains speculative due to the overlapping specificity of proteases and the presence of less specific proteases. Nevertheless, the observed associations reveal signatures of endoproteolytic and exoproteolytic degradation of proteins and direct future studies in further investigation of the role of these two mechanisms in shaping the human milk peptidome.

### Associations between proteins and peptides

We found that from all identified proteins with potential protease activity (*n* = 25), only 9 appeared in a cluster with peptides. The most probable explanation for the lack of strong associations between proteases and peptide clusters is the fact that the abundance of a protease is not necessarily equal to or related to its proteolytic activity in the natural milk environment. This can be due to, for example, the protease being present in the zymogen or inactive state, the pH of the milk, or the inhibition of proteases through protease inhibitors. Although most of the observed associations are between molecular features of the same type, that is, among proteins and among peptides, several interesting associations were found between proteins and peptides and will be discussed.

**Figure 2:**
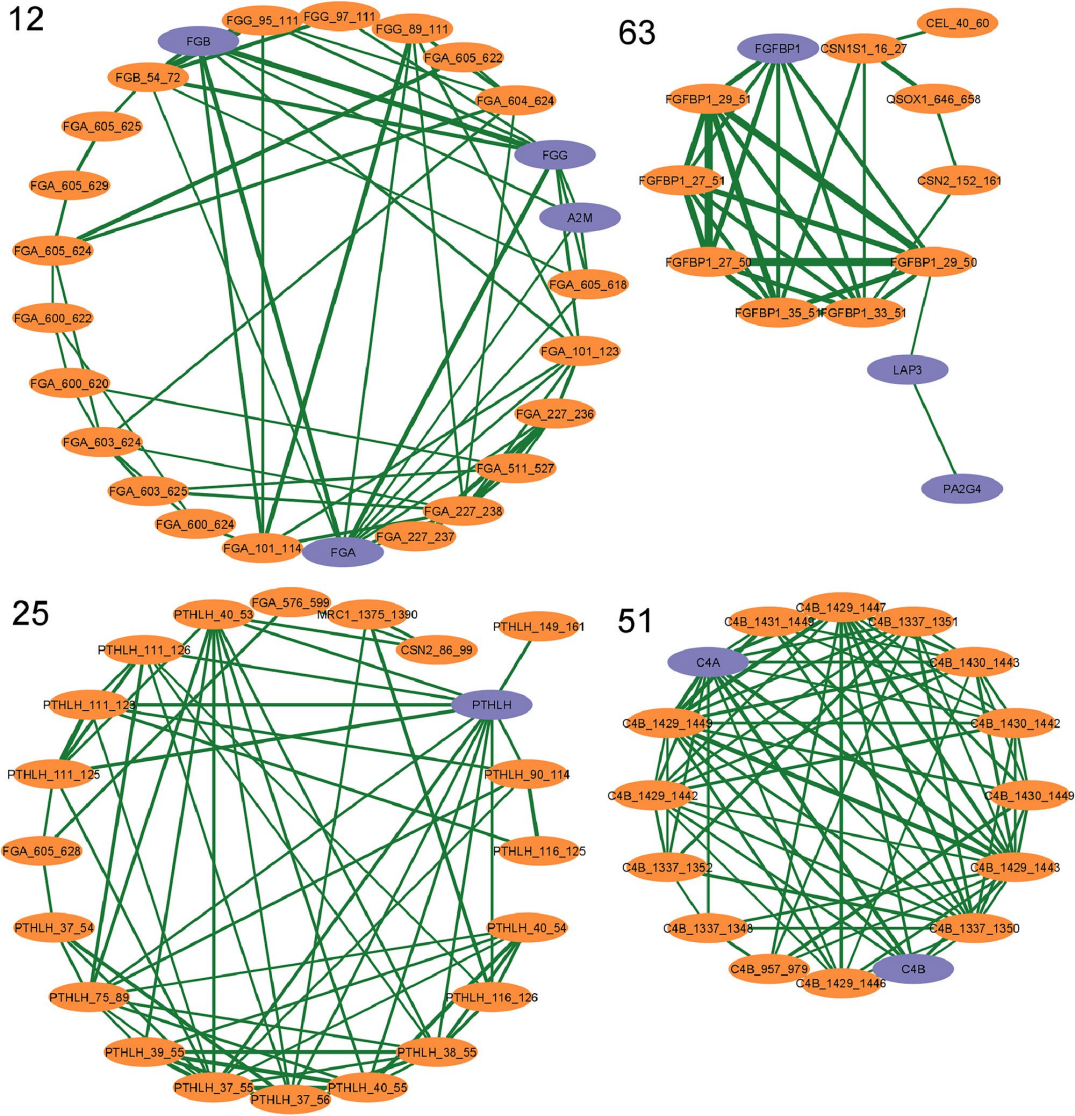
Network representation (circular layout) of a selection of clusters. The network is constructed by calculation of Gaussian graphical models (GGMs) and subsequent clustering with the Leiden community detection algorithm. Purple nodes represent proteins with their respective gene names, and orange nodes represent peptides with the gene name of the precursor protein and the respective sequence range. The thickness of the edges is proportional to the partial correlation coefficients from the GGMs. Selected clusters show associations between proteins and peptides and the selection of clusters is made from Fig. 1 with corresponding cluster labels.

We found that the fibrinogen chains that make up the fibrinogen complex ($\alpha$ (FGA), $\beta$ (FGB), and $\gamma$ (FGG)), associated strongly with fibrinogen peptides (Fig. 2 Cluster 12). Fibrinogen is a protein complex synthesized in the liver, which plays a central role in blood coagulation. This coagulation is activated when fibrinopeptides are cleaved off enzymatically from both FGA and FGB by thrombin, resulting in the formation of fibrin and fibrin clots^48^. The fibrinopeptide of FGA was identified in the peptide data, suggesting that fibrinogen chains present in human milk can occur in the activated form, that is, as fibrin. To prevent clot formation, fibrin is degraded by PLG^49^, a process referred to as fibrinolysis. It is known that active PLG is present in human milk^50^, and several of the degradation products formed during fibrinolysis can be observed in Cluster 12 in Fig. 2^51^. Furthermore, from all cleavage sites of the fibrinogen peptides, 50% matches the specificity of plasmin. It can be noted that FGA is more degraded, with 28 identified peptides, whereas FGB and FGG have 1 and 3 identified peptides, respectively. This matches with the fact that the FGA chain is cleaved first in the degradation of fibrin^52^. Additionally, $\alpha$-2-macroglobulin (A2M), a protease inhibitor that is known to regulate the degradation of fibrin by inhibition of PLG, also appears in Cluster 12. These findings indicate the presence and association of several components and degradation products of blood coagulation in human milk. The origin of these proteins and peptides remains an open question. One explanation might be that they are blood-derived and indirectly end up in the milk through, for example, damage of the mammary epithelial barrier. Nevertheless, it is more probable that they are part of the standard human milk composition since FGA was identified in 296 of the 297 samples. This also agrees with a study by Green et al., who investigated PLG-deficient mice and suggested that an accumulation of fibrin in the mammary gland could block mammary ducts and ultimately induce involution^53^. Our observation of positive associations between fibrinogen chains and their degradation products suggests that if more fibrinogen is present in the milk, more degradation occurs. From this, it can be hypothesized that the fibrinolysis pathway in milk is present to prevent blocked ducts and, therefore, to maintain lactation. Further research is required to identify the determinants of the presence of these proteins and peptides in human milk.

Cluster 25 comprises, among others, parathyroid hormone-related protein (PTHLH, also known as PTHRP) and 16 of its peptides. It has been suggested that PTHLH is involved in regulating calcium transport through the mammary gland^54^. After synthesis, PTHLH is degraded into three secretory forms, ranging from sequence position 37–72, 74–130, 143–175, respectively (signal peptide is included in the numbering of the sequence positions)^55^. It can be noted from Cluster 25 in Fig. 2 that peptides derived from all three secretory forms of PTHLH were identified and associated with the precursor protein. Although the functions and determinants of the different secretory forms of PTHLH in human milk are not known yet, our results show that they are all present in the secretory form in the milk and that their abundance depends on the abundance of intact PTHLH.

Cluster 51 (Fig. 2) shows the association between peptides from complement component 4 (C4) and the intact C4 isotypes C4A and C4B. These proteins are part of the complement system, a set of proteins, enzymes, and receptors in the blood that plays a key role in the innate immune system’s defense against pathogens. Several other proteins from this complex were identified in the proteomics data, including C3, C7, C9, plasma protease C1 inhibitor (SERPING1), and complement factor H (CFH). The presence of complement proteins in human milk has been evidenced before^56^ and can boost the protective mechanisms of the infants’ mucosae^57^. A recent study by Xu et al., showed in mice that the presence of complement components in milk plays an important role in modification of the gut microbiota and subsequently lysis of bacterial cells^58^. Nevertheless, it remains unknown what determines the abundance of these proteins and peptides in human milk, and further research is needed to examine this further. The identification of C4 in the current study (sequence coverage = 84%) covers regions between sequence positions 23 and 1682, whereas the total length of C4 is 1744 amino acids. This provides evidence for the presence of intact C4 in human milk. C4 can participate in the classical and lectin complement pathways and is cleaved into fragments upon activation^59^. In the peptide fraction, all but one of the C4 peptides that were identified originate from a specific region (between positions 1337 and 1449), which is the C-terminal part of the C4b fragment (position 757–1446). This C-terminal region of C4b is cleaved off in the formation of the C4d fragment (position 957–1336). Together, this shows that the identified C4 peptides are byproducts of the activation cascade of C4^51^. The association of these peptides with intact C4 suggests that the presence of activated fragments of C4 in human milk depends on the abundance of intact C4.

Fibroblast growth factor-binding protein 1 (FGFBP1) is a protein that can bind fibroblast growth factors (FGFs), a family of cell signaling proteins, and release them from the extracellular matrix. All identified FGFBP1 peptides (*n* = 6) originate from the N-terminal region of the protein (between positions 24 and 51), which is also covered in the identification of the intact protein. Of the cleavage sites of the peptides, 15 out of 24 have lysine in position P1, suggesting that PLG is responsible for most of the cleavages. The strong association between the peptides and their intact protein (Fig. 2 Cluster 63) suggests a specific proteolytic degradation unrelated to the degradation of other proteins in milk. Such degradation might be related to the role of FGFBP1 in protecting FGF against degradation^60^, but this remains speculative since no previous studies were found on proteolytic degradation of FGFBP1.

Overall, the protein-peptide associations revealed several mechanisms of specific proteolytic degradation that take place in human milk. Specifically, degradation of fibrin(ogen), PTHLH, complement C4, and FGFBP1 was associated with the abundance of their precursor proteins. The degree of proteolysis of these proteins differs from the proteolytic degradation of the most abundant precursor proteins in milk.

## Methods

### Experimental design and statistical rationale

Human milk samples from a selection of 300 mother-child dyads from the CHILD Cohort Study were used. The selection of these samples was made based on the allergy status of the mother and the infant, including equal numbers of different combinations of mother-child allergy statuses^61^. The information on allergy status was used in a previous investigation where specifically the relation between allergy status and the milk proteome was investigated^61^. In the current study the allergy status was not used in the data analysis. During the data analysis, 3 samples were omitted as outliers due to their distinct peptide profile. These samples showed a total peptide abundance several magnitudes higher than the average, possibly due to the occurrence of mastitis. The reported results concern therefore 297 samples.

Samples were analyzed in randomized order, with a technical replicate added randomly to every 7 injections. In addition, technical replicates were added as a control for technical variation and were prepared from a pooled human milk sample from the Dutch Human Milk Bank (Amsterdam, The Netherlands).

### Sample collection and handling

The CHILD Cohort Study is a Canadian national population-based cohort (https://www.childstudy.ca) in which information was collected over time from parents and their infants^62^. Pregnant mothers were recruited from the general population from Vancouver, Edmonton, Manitoba, and Toronto. Local Human Research Ethics Boards approved the study protocols, and the study was carried out following the Declaration of Helsinki. All parents provided written informed consent at the time of enrollment in the study. Milk samples were collected according to the CHILD protocol^63^. In short, foremilk and hindmilk samples expressed prior to and after feeding the infant were collected from several feedings during a day and were pooled to minimize within-feed variation and diurnal variation. Samples were collected between 6 and 35 weeks post-partum [median = 15.6 weeks, interquartile range (IQR) = 4.6]. Samples were stored at 4 ^∘^C in the home refrigerator and, within 24 hours, picked up and transported on ice to the CHILD laboratory. There, samples were aliquoted and stored until further analysis at − 80 ^∘^C. Further transport of the samples was done on dry ice. Temporal storage of the samples at 4 ^∘^C allowed for post-expression protein degradation. However, post-synthesis protein degradation takes place within the mammary gland as well as before and after expression^5^. Milk with proteins partially degraded post-synthesis therefore reflects what the infant consumes. Furthermore, a study by Howland et al., showed that differences among individual mothers can still be detected after temporal storage at 4 ^∘^C^64^.

### Proteomics

The proteomics data used in this manuscript has been described in a previous manuscript^61^. In the current study, a stricter filtering of the data on missing values was applied. To aid the reader, we provide here a brief description of the sample preparation and analysis.

#### Sample preparation

Skimmed milk was obtained by centrifugation at 10,000*g* and 4 ^∘^C for 30 min. Then, skimmed milk was again centrifuged at 1000*g* and 4 ^∘^C for 10 min to remove any remaining lipids. Finally, skimmed milk samples were prepared with filter-aided sample preparation for protein analysis as described before^20^.

#### LC-MS/MS analysis

Trypsin digested proteins were analyzed with LC-MS/MS as described before, with minor adjustments^65^.

#### Data processing

The Andromeda search engine of the MaxQuant software v1.6.17.0 was used to analyze the raw LC-MS/MS data^66^. A database with protein sequences was created by an initial MaxQuant run using the full human proteome (downloaded from UniProtKB on 20-01-2021, *n* = 194,237)^67^. Protein identifiers obtained as identification from this initial run were used to create a human milk database for a second run (*n* = 24,175), in which also a cow milk protein (*n* = 1006) and an allergen protein database (*n* = 721) were added^68^. A full description of how these databases were obtained can be found in Dekker et al.^68^.

In MaxQuant, digestion specificity was set to Trypsin/P, with maximally 2 missed cleavages. A fixed propionamide modification was set for cysteines, and variable modifications for acetylation of the peptide N-term, deamidation of the side chains of asparagine and glutamine, and oxidation of methionine, with a maximum of 5 modifications per peptide were set. A leading protein was selected for each identified protein group as described elsewhere^68^. A false discovery rate of 1% was used at both peptide and protein levels. The first search had 20 ppm peptide tolerance, the main search 4.5 ppm tolerance, and the MS/MS fragment mass tolerance was 20 ppm. Label-free quantification (LFQ) was used to obtain protein abundances. Gene names were used to abbreviate protein names where appropriate.

### Peptidomics

#### Sample preparation

Skimmed milk samples were prepared for peptide analysis as previously described^20^. In short, proteins were removed using precipitation. For this, an equal volume of 200 g/L trichloroacetic acid in milli-Q water was added, followed by centrifugation at 3000*g* for 10 min at 4 ^∘^C. From the supernatant that was obtained, 50 $\upmu$L was cleaned up using solid phase extraction (SPE) on C18+ Stage tip columns (prepared in-house), as previously described^19,69^. Finally, eluted peptides were reconstituted in 50 $\upmu$L of 1 mL/L formic acid in water.

#### LC-MS/MS analysis

Peptides were analyzed with LC-MS/MS, using the same method as for the protein analysis described above. For the peptidomics analysis, 4 $\upmu$L of peptide solution was loaded onto the column.

#### Data processing

The raw LC-MS/MS data files from the peptide analysis were processed similarly to the proteomics data. Differences were the digestion specificity which was set to unspecific without fixed cysteine modification and with variable modifications for acetylation of the protein N-term, deamidation of the side chains of asparagine and glutamine, and oxidation of methionine, with a maximum of 5 modifications per peptide. The sequence database which was created for the processing of the proteomics data containing human milk, cow milk, and allergen proteins, was used (as described above). Peptide length was set to a minimum of 8 and a maximum of 25 amino acids, to achieve the best compromise between computational time and complete identification. It was shown in a study by Dingess et al., that the majority of the peptides endogenously present in human milk are covered by this range in peptide length^70^. Raw intensities were used for further data analysis.

### Statistical methods

Statistical analysis and visualizations were, unless specified differently, carried out using R version 4.0.1^71^.

#### Missing data

MaxQuant proteinGroups (proteomics) or peptides (peptidomics) result files were filtered so that common contaminants were removed and only proteins and peptides that were identified in more than half (>150) of the samples were retained. In this way, a selection of the most prevalent and abundant proteins and peptides was used for further data analysis. Outliers (*n* = 3) were removed and in the remaining data, missing values were imputed using the GSimp package for R with default parameters^72^, which implements a Gibbs sampler-based algorithm to impute missing values with the assumption that missing values are not at random (MNAR) and left censored.

#### Graphical Gaussian modeling and network analysis

To investigate associations within and between the datasets, network analysis was applied to a combined data matrix comprising proteins (*n* = 456) and peptides (*n* = 1455) in 297 samples.

To build the network, partial correlations were estimated using Gaussian graphical modeling (GGM). The GGMs were built with a shrinkage-based regularization approach, which estimates the partial correlation coefficients in a pairwise manner. To build the GGMs, the *ggm.estimator.pcor* function from the GeneNet package for R was used^71,73^.

Partial correlation coefficients $\rho$_ij_ describe the pairwise correlation between protein or peptide *X*_i_ and *X*_j_ after accounting for their correlation with all other proteins and peptides. This approach accounts for confounders and covariates, indirect associations often present in omics data sets, and enabled the study of direct associations among proteins, among peptides, and between proteins and peptides.

In the inference of the network, only significant edges were used. To determine the significance of the edges, the built-in empirical Bayes local false discovery rate (fdr) statistic was used^74^. Edges were considered significant if the probability of their “presence” was larger than 0.9 (which is equal to a local fdr < 0.1).

#### Network visualization and clustering

Adjacency matrices with partial correlations from the GGMs were visualized in networks using Cytoscape v3.9.1^75^. In the GGM network, proteins and peptides are presented as nodes, and GGM-estimated partial correlations are the

edges between the nodes. Subsequent clustering of networks was performed using the Leiden algorithm^76^, through the clusterMaker2 plugin for Cytoscape^77^. For this clustering, Constant Potts Model was used as a quality function with a resolution parameter of 10^-3^, $\beta$ value 0.01, and 1000 iterations. Clusters comprising more than 3 nodes were retained for further investigation.

#### Overrepresentation analysis

To determine whether protein clusters were overrepresented with specific gene ontology (GO) annotations, the GORILLA tool (Gene Ontology enRIchment anaLysis and visuaLizAtion tool) (http://cbl-gorilla.cs.technion.ac.il/)^78^ was used. The two-list mode was used, with all identified proteins as the background set. *p* values were corrected with the Benjamini–Hochberg method^79^. An adjusted *p* value < 0.05 was considered significant.

### Supplementary Information

Supplementary Figure S1.

Supplementary Table S1.

Supplementary Table S2.

Supplementary Table S3.

Supplementary Table S4.

## Data Availability

The mass spectrometry proteomics and peptidomics data have been deposited to the ProteomeXchange Consortium via the PRIDE^80^ partner repository with the data set identifiers PXD034806 and PXD036477. Sample metadata can be made available upon request. Requests can be submitted via email to child@mcmaster.ca.
