# Supplementary figures and images for: Network analysis of the proteome and peptidome sheds light on human milk as a biological system
Source: Sci Rep. 2024 Mar 30;14:7569. doi: 10.1038/s41598-024-58127-2 (PMC10981717; doi:10.1038/s41598-024-58127-2)

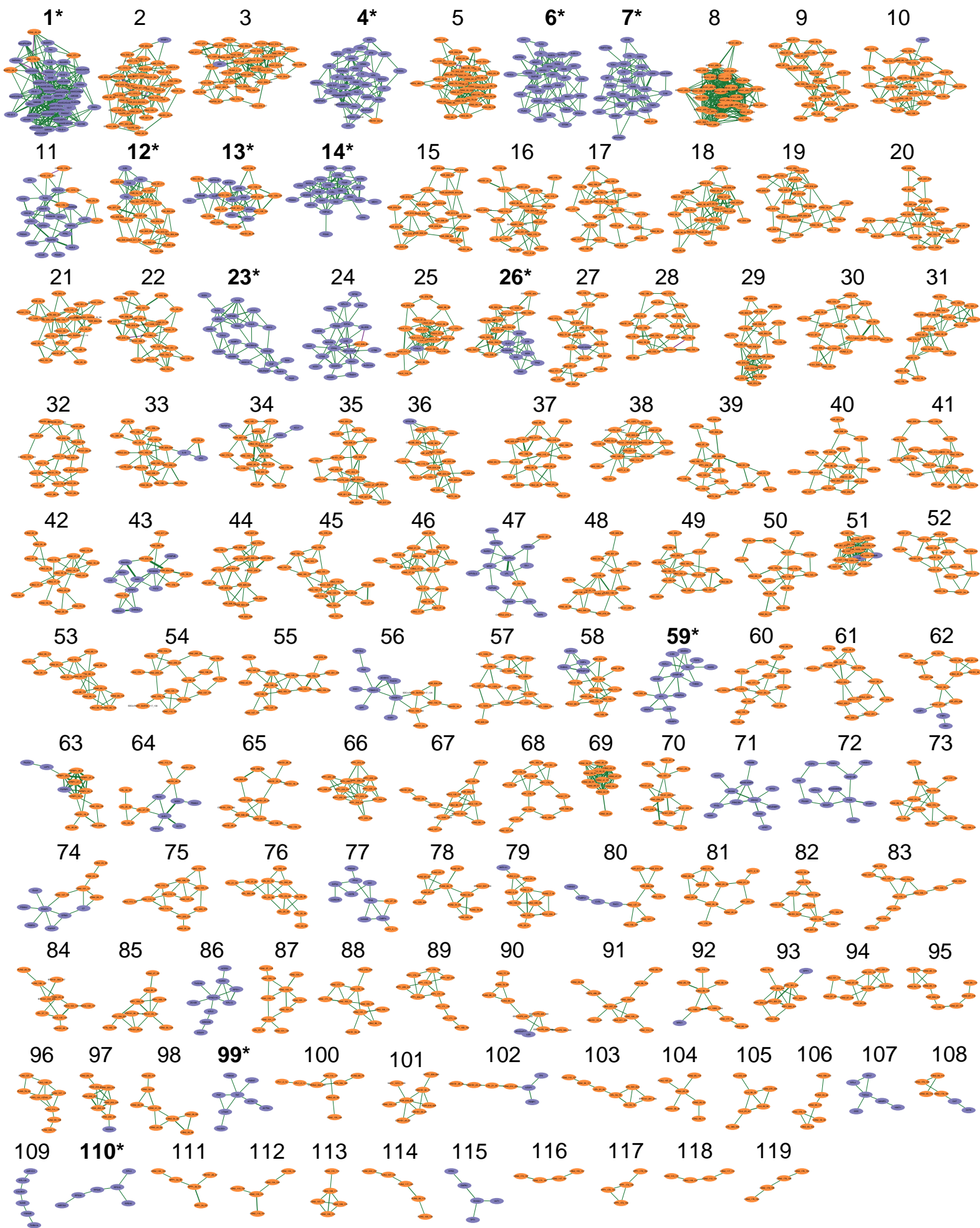

Supplement: Supplementary file 1 — Supplementary Figure S1. [file 41598_2024_58127_MOESM1_ESM.pdf]
